# Bis[2-bromo-4-(2-hy­droxy­eth­yl)phenol] monohydrate

**DOI:** 10.1107/S160053681101066X

**Published:** 2011-04-13

**Authors:** Jing Zhu, Wen-ge Yang, Lu-lu Wang, Kai Wang, Yong-hong Hu

**Affiliations:** a State Key Laboratory of Materials-Oriented Chemical Engineering, School of Pharmaceutical Sciences, Nanjing University of Technology, Xinmofan Road No. 5, Nanjing 210009, People’s Republic of China; b State Key Laboratory of Materials-Oriented Chemical Engineering, College of Life Science and Pharmaceutical Engineering, Nanjing University of Technology, Xinmofan Road No. 5, Nanjing 210009, People’s Republic of China

## Abstract

In the title compound, 2C_8_H_9_BrO_2_·H_2_O, the O—C—C—C torsion angles for the hy­droxy­ethyl group and the Br—C—C—O torsion angles involving bromo and phenol groups are 61.7 (11) and 0.7 (12)°, respectively, in one independent mol­ecule and 61.5 (11) and 0.2 (11)°, respectively, in the other. In the crystal, mol­ecules are linked through O—H⋯O and O—H⋯Br hydrogen bonds, forming a polymeric chain.

## Related literature

For synthesis of the title compound and background information, see: Bovicelli *et al.* (2007 ▶). For a related structure, see: Mewett *et al.* (2009 ▶).
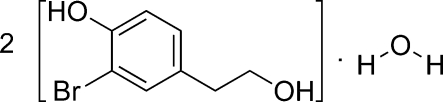


## Experimental

### 

#### Crystal data

2C_8_H_9_BrO_2_·H_2_O
*M* *_r_* = 452.14
Monoclinic, 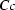

*a* = 5.9790 (12) Å
*b* = 18.396 (4) Å
*c* = 16.801 (3) Å
β = 98.83 (3)°
*V* = 1826.0 (6) Å^3^
*Z* = 4
Mo *K*α radiation
μ = 4.46 mm^−1^
*T* = 293 K
0.20 × 0.10 × 0.10 mm

#### Data collection

Enraf–Nonius CAD-4 diffractometer
Absorption correction: ψ scan (ψ-scans; North *et al.*, 1968 ▶) *T*_min_ = 0.469, *T*_max_ = 0.664
3585 measured reflections
1827 independent reflections
1302 reflections with *I* > 2σ(*I*)
*R*_int_ = 0.045
3 standard reflections every 200 reflections  intensity decay: 1%

#### Refinement

*R*[*F* ^2^ > 2σ(*F* ^2^)] = 0.045
*wR*(*F* ^2^) = 0.095
*S* = 1.01
1827 reflections
208 parameters
2 restraints
H-atom parameters constrained
Δρ_max_ = 0.32 e Å^−3^
Δρ_min_ = −0.45 e Å^−3^
Absolute structure: Flack (1983 ▶), 746 Friedel pairs
Flack parameter: 0.00 (2)

### 

Data collection: *CAD-4 EXPRESS* (Enraf–Nonius, 1989 ▶); cell refinement: *CAD-4 EXPRESS*; data reduction: *XCAD4* (Harms & Wocadlo, 1995 ▶); program(s) used to solve structure: *SHELXS97* (Sheldrick, 2008 ▶); program(s) used to refine structure: *SHELXL97* (Sheldrick, 2008 ▶); molecular graphics: *SHELXTL* (Sheldrick, 2008 ▶); software used to prepare material for publication: *PLATON* (Spek, 2009 ▶).

## Supplementary Material

Crystal structure: contains datablocks global, I. DOI: 10.1107/S160053681101066X/pv2391sup1.cif

Structure factors: contains datablocks I. DOI: 10.1107/S160053681101066X/pv2391Isup2.hkl

Additional supplementary materials:  crystallographic information; 3D view; checkCIF report

## Figures and Tables

**Table 1:** Hydrogen-bond geometry (Å, °)

*D*—H⋯*A*	*D*—H	H⋯*A*	*D*⋯*A*	*D*—H⋯*A*
O*W*—H*WA*⋯Br1	0.89	2.86	3.537 (8)	134
O*W*—H*WA*⋯O2	0.89	2.00	2.820 (10)	151
O1—H1*A*⋯O4	0.82	1.84	2.633 (9)	163
O*W*—H*WB*⋯O1^i^	0.88	2.07	2.745 (10)	133
O2—H2*A*⋯Br1	0.85	2.56	3.026 (6)	115
O2—H2*A*⋯O*W*	0.85	2.18	2.820 (9)	131
O3—H3*A*⋯O2^ii^	0.82	1.80	2.592 (9)	162
O4—H4*B*⋯Br2	0.85	2.57	3.039 (7)	116
O4—H4*B*⋯O*W*^iii^	0.85	2.21	2.804 (10)	127

Symmetry codes: (i) 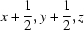; (ii) 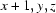; (iii) 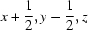.

**Fig. 1.**
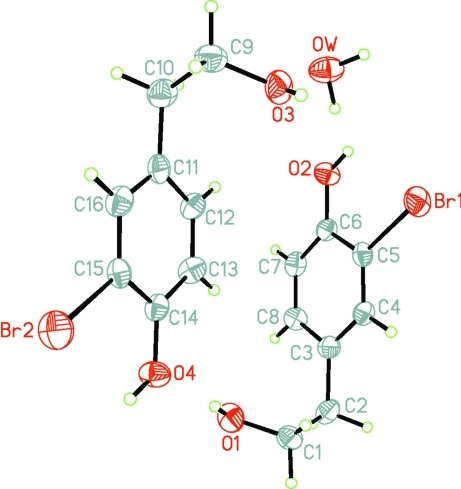
The molecular structure of the title molecule, with the atom numbering scheme. Displacement ellipsoids are drawn at 30% probability levels.

**Fig. 2.**
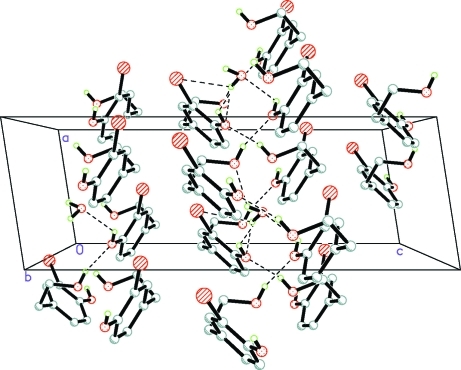
A practical packing diagram of the title compound. Hydron bonds are shown as dashed lines.

## supplementary crystallographic information

### Comment

The title compound is used as the key intermediate in the synthesis of hydroxytyrosol (Bovicelli *et al.*, 2007). As a part of our studies on the synthesis of hydroxytyrosol we report herein the crystal structure of the title compound.

In the molecules of the title compound, (Fig. 1), the bond lengths  and angles agree very well with the corresponding bond lengths and angles reporeted in a structure containing the title compound as its fragment (Mewett *et al.*, 2009).

In the crystal structure, O—H···O and O—H···Br type hydrogen bonding interactions (Table 1) link the molecules into ribbons extended along the *a*-axis (Fig. 2). The two molecules of the title compound in the asymmetric unit are identical. The torsion angles O1/C1/C2/C3 and C1/C2/C3/C8 are 61.7 (12) and -100.6 (11)°, respectively, in one molecule.  The corresponding torion angles in the other molecule are 61.5 (11) and -101.5 (11)° (for O3/C9/C10/C11 and C9/C10/C11/C12, respectively).

### Experimental

To a solution of 4-hydroxyphenethyl alcohol (217.4 mmol, 30 g) and NaBr (217.4 mmol, 22.17 g) in acetone (600 ml), a solution of oxone (200 g) in water (1 L) was added dropwise at 263 K within 3 h. The progress of the reaction was monitored by thin-layer chromatography (TLC, hexane/ethyl acetate 6:4), and when the reaction was over (complete consumption of the substrate), AcOEt (500 ml) was added to the mixture. The organic layer was separated, and the aqueous phase was extracted with two 300 mL portions of AcOEt. The combined organic solutions were washed with water (300 ml), dried over anhydrous Na_2_SO_4_ (100 g), and evaporated. The monobrominated product, obtained in almost quantitative yield (47.1 g), appeared to be spectroscopically pure, white solid (Bovicelli *et al.*, 2007). Crystals of (I) suitable for X-ray diffraction were obtained by slow evaporation of an ethanol solution.

### Refinement

H atoms were positioned geometrically with C—H = 0.93, 0.98 and 0.97 Å for aromatic, methine and methylene H atoms, respectively, and constrained to ride on their parent atoms, with *U*_iso_(H) = 1.2 (or 1.5 for methyl groups) times *U*_eq_(C).

### Crystal data

**Table:**

2C_8_H_9_BrO_2_·H_2_O	*F*(000) = 904
*M**_r_* = 452.14	*D*_x_ = 1.645 Mg m^−^^3^
Monoclinic, *C**c*	Mo *K*α radiation, λ = 0.71073 Å
Hall symbol: C -2yc	Cell parameters from 25 reflections
*a* = 5.9790 (12) Å	θ = 9–13°
*b* = 18.396 (4) Å	µ = 4.46 mm^−^^1^
*c* = 16.801 (3) Å	*T* = 293 K
β = 98.83 (3)°	Block, colorless
*V* = 1826.0 (6) Å^3^	0.20 × 0.10 × 0.10 mm
*Z* = 4	

### Data collection

**Table:**

Enraf–Nonius CAD-4 diffractometer	1302 reflections with *I* > 2σ(*I*)
Radiation source: fine-focus sealed tube	*R*_int_ = 0.045
graphite	θ_max_ = 25.3°, θ_min_ = 2.2°
ω/2θ scans	*h* = 0→7
Absorption correction: ψ scan (ψ-scans; North *et al.*, 1968)	*k* = −22→22
*T*_min_ = 0.469, *T*_max_ = 0.664	*l* = −20→19
3585 measured reflections	3 standard reflections every 200 reflections
1827 independent reflections	intensity decay: 1%

### Refinement

**Table:**

Refinement on *F*^2^	Secondary atom site location: difference Fourier map
Least-squares matrix: full	Hydrogen site location: inferred from neighbouring sites
*R*[*F*^2^ > 2σ(*F*^2^)] = 0.045	H-atom parameters constrained
*wR*(*F*^2^) = 0.095	*w* = 1/[σ^2^(*F*_o_^2^) + (0.050*P*)^2^] where *P* = (*F*_o_^2^ + 2*F*_c_^2^)/3
*S* = 1.01	(Δ/σ)_max_ < 0.001
1827 reflections	Δρ_max_ = 0.32 e Å^−^^3^
208 parameters	Δρ_min_ = −0.45 e Å^−^^3^
2 restraints	Absolute structure: Flack (1983), 746 Friedel pairs
Primary atom site location: structure-invariant direct methods	Flack parameter: 0.00 (2)

### Special details

**Table:**

Geometry. All e.s.d.'s (except the e.s.d. in the dihedral angle between two l.s. planes) are estimated using the full covariance matrix. The cell e.s.d.'s are taken into account individually in the estimation of e.s.d.'s in distances, angles and torsion angles; correlations between e.s.d.'s in cell parameters are only used when they are defined by crystal symmetry. An approximate (isotropic) treatment of cell e.s.d.'s is used for estimating e.s.d.'s involving l.s. planes.
Refinement. Refinement of *F*^2^ against ALL reflections. The weighted *R*-factor *wR* and goodness of fit *S* are based on *F*^2^, conventional *R*-factors *R* are based on *F*, with *F* set to zero for negative *F*^2^. The threshold expression of *F*^2^ > σ(*F*^2^) is used only for calculating *R*-factors(gt) *etc*. and is not relevant to the choice of reflections for refinement. *R*-factors based on *F*^2^ are statistically about twice as large as those based on *F*, and *R*- factors based on ALL data will be even larger.

### Fractional atomic coordinates and isotropic or equivalent isotropic displacement parameters (Å^2^)

**Table:**

	*x*	*y*	*z*	*U*_iso_*/*U*_eq_	
Br2	0.98869 (14)	0.25839 (6)	0.74826 (7)	0.0700 (4)	
O3	0.7384 (12)	0.5738 (3)	0.6248 (4)	0.0621 (18)	
H3A	0.8398	0.5653	0.5983	0.093*	
O4	0.5409 (11)	0.2412 (3)	0.6368 (4)	0.0566 (17)	
H4B	0.6442	0.2103	0.6523	0.068*	
C9	0.827 (2)	0.5705 (6)	0.7086 (6)	0.060 (3)	
H9A	0.8513	0.6194	0.7299	0.072*	
H9B	0.9718	0.5455	0.7161	0.072*	
C10	0.6614 (18)	0.5301 (5)	0.7535 (6)	0.061 (3)	
H10A	0.7193	0.5299	0.8107	0.073*	
H10B	0.5173	0.5554	0.7458	0.073*	
C11	0.6262 (19)	0.4526 (5)	0.7238 (6)	0.053 (3)	
C12	0.4362 (16)	0.4334 (5)	0.6716 (5)	0.048 (2)	
H12A	0.3248	0.4680	0.6560	0.058*	
C13	0.4082 (16)	0.3643 (5)	0.6423 (5)	0.051 (2)	
H13A	0.2781	0.3530	0.6066	0.062*	
C14	0.5678 (14)	0.3105 (5)	0.6643 (5)	0.044 (2)	
C15	0.7609 (14)	0.3305 (5)	0.7164 (5)	0.042 (2)	
C16	0.7941 (16)	0.3996 (5)	0.7461 (6)	0.052 (2)	
H16A	0.9260	0.4113	0.7807	0.062*	
OW	0.3419 (11)	0.6470 (4)	0.5757 (5)	0.074 (2)	
HWA	0.2649	0.6129	0.5450	0.089*	
HWB	0.3857	0.6750	0.5383	0.089*	
Br1	0.34194 (14)	0.53555 (5)	0.40416 (7)	0.0640 (3)	
O1	0.1966 (11)	0.2167 (3)	0.5222 (4)	0.064 (2)	
H1A	0.3182	0.2261	0.5501	0.096*	
C1	0.2190 (18)	0.2218 (6)	0.4413 (6)	0.054 (3)	
H1B	0.2241	0.1734	0.4187	0.065*	
H1C	0.3597	0.2463	0.4361	0.065*	
O2	−0.0093 (10)	0.5504 (3)	0.5150 (4)	0.0542 (17)	
H2A	0.0843	0.5814	0.5021	0.065*	
C2	0.0224 (18)	0.2636 (5)	0.3950 (6)	0.058 (3)	
H2B	0.0376	0.2644	0.3383	0.069*	
H2C	−0.1178	0.2388	0.4002	0.069*	
C3	0.0112 (17)	0.3401 (5)	0.4246 (5)	0.049 (2)	
C4	0.1511 (15)	0.3940 (5)	0.4051 (5)	0.044 (2)	
H4A	0.2537	0.3834	0.3703	0.053*	
C5	0.1449 (15)	0.4620 (5)	0.4346 (5)	0.045 (2)	
C6	−0.0025 (16)	0.4810 (5)	0.4867 (5)	0.044 (2)	
C7	−0.1492 (16)	0.4280 (5)	0.5058 (5)	0.051 (2)	
H7A	−0.2543	0.4396	0.5393	0.061*	
C8	−0.1427 (15)	0.3594 (5)	0.4767 (6)	0.050 (2)	
H8A	−0.2415	0.3246	0.4914	0.060*	

### Atomic displacement parameters (Å^2^)

**Table:**

	*U*^11^	*U*^22^	*U*^33^	*U*^12^	*U*^13^	*U*^23^
Br2	0.0482 (5)	0.0791 (8)	0.0764 (7)	0.0122 (6)	−0.0106 (5)	0.0016 (7)
O3	0.073 (5)	0.066 (4)	0.054 (4)	0.009 (4)	0.032 (4)	0.004 (3)
O4	0.045 (4)	0.050 (4)	0.072 (4)	0.009 (4)	−0.002 (3)	−0.008 (4)
C9	0.070 (7)	0.047 (6)	0.066 (7)	−0.003 (5)	0.018 (6)	−0.007 (5)
C10	0.064 (7)	0.064 (7)	0.058 (6)	−0.005 (6)	0.019 (5)	−0.007 (5)
C11	0.065 (7)	0.058 (6)	0.039 (6)	−0.006 (6)	0.017 (5)	0.010 (5)
C12	0.038 (5)	0.060 (6)	0.046 (5)	0.001 (5)	0.002 (4)	−0.004 (5)
C13	0.033 (5)	0.062 (7)	0.057 (6)	0.002 (5)	0.001 (4)	−0.008 (5)
C14	0.035 (5)	0.056 (6)	0.040 (5)	−0.008 (4)	0.001 (4)	0.000 (4)
C15	0.032 (5)	0.051 (6)	0.043 (5)	0.002 (4)	0.004 (4)	0.009 (4)
C16	0.043 (6)	0.066 (7)	0.046 (6)	−0.009 (5)	0.005 (4)	−0.001 (5)
OW	0.059 (5)	0.071 (4)	0.095 (5)	−0.010 (4)	0.020 (4)	−0.024 (4)
Br1	0.0591 (6)	0.0682 (6)	0.0715 (7)	−0.0019 (6)	0.0322 (5)	0.0040 (6)
O1	0.054 (4)	0.067 (4)	0.062 (4)	−0.012 (4)	−0.020 (4)	0.005 (3)
C1	0.054 (7)	0.049 (6)	0.058 (7)	0.015 (5)	0.004 (5)	0.003 (5)
O2	0.043 (4)	0.050 (4)	0.074 (4)	−0.005 (3)	0.022 (3)	−0.002 (3)
C2	0.059 (7)	0.069 (7)	0.045 (6)	0.007 (6)	0.006 (5)	−0.009 (5)
C3	0.051 (6)	0.048 (6)	0.044 (6)	0.005 (5)	−0.008 (5)	−0.001 (5)
C4	0.041 (5)	0.053 (6)	0.039 (5)	−0.001 (5)	0.005 (4)	−0.001 (5)
C5	0.044 (5)	0.051 (5)	0.042 (5)	−0.004 (4)	0.009 (4)	0.006 (5)
C6	0.039 (5)	0.051 (7)	0.042 (5)	0.003 (5)	0.009 (4)	−0.002 (5)
C7	0.038 (5)	0.067 (7)	0.050 (5)	0.012 (5)	0.013 (4)	0.003 (5)
C8	0.037 (5)	0.053 (6)	0.059 (6)	0.001 (5)	0.007 (5)	0.001 (5)

### Geometric parameters (Å, °)

**Table:**

Br2—C15	1.918 (8)	OW—HWB	0.8827
O3—C9	1.428 (11)	Br1—C5	1.915 (9)
O3—H3A	0.8200	O1—C1	1.391 (12)
O4—C14	1.357 (11)	O1—H1A	0.8200
O4—H4B	0.8500	C1—C2	1.515 (14)
C9—C10	1.529 (14)	C1—H1B	0.9700
C9—H9A	0.9700	C1—H1C	0.9700
C9—H9B	0.9700	O2—C6	1.365 (10)
C10—C11	1.515 (13)	O2—H2A	0.8500
C10—H10A	0.9700	C2—C3	1.497 (12)
C10—H10B	0.9700	C2—H2B	0.9700
C11—C12	1.370 (14)	C2—H2C	0.9700
C11—C16	1.409 (14)	C3—C4	1.370 (12)
C12—C13	1.364 (12)	C3—C8	1.410 (13)
C12—H12A	0.9300	C4—C5	1.348 (11)
C13—C14	1.385 (12)	C4—H4A	0.9300
C13—H13A	0.9300	C5—C6	1.380 (12)
C14—C15	1.388 (11)	C6—C7	1.382 (12)
C15—C16	1.368 (12)	C7—C8	1.355 (12)
C16—H16A	0.9300	C7—H7A	0.9300
OW—HWA	0.8938	C8—H8A	0.9300
			
C9—O3—H3A	109.5	C1—O1—H1A	109.5
C14—O4—H4B	118.7	O1—C1—C2	110.7 (8)
O3—C9—C10	109.7 (8)	O1—C1—H1B	109.5
O3—C9—H9A	109.7	C2—C1—H1B	109.5
C10—C9—H9A	109.7	O1—C1—H1C	109.5
O3—C9—H9B	109.7	C2—C1—H1C	109.5
C10—C9—H9B	109.7	H1B—C1—H1C	108.1
H9A—C9—H9B	108.2	C6—O2—H2A	118.8
C11—C10—C9	111.3 (8)	C3—C2—C1	112.2 (8)
C11—C10—H10A	109.4	C3—C2—H2B	109.2
C9—C10—H10A	109.4	C1—C2—H2B	109.2
C11—C10—H10B	109.4	C3—C2—H2C	109.2
C9—C10—H10B	109.4	C1—C2—H2C	109.2
H10A—C10—H10B	108.0	H2B—C2—H2C	107.9
C12—C11—C16	118.6 (9)	C4—C3—C8	116.5 (8)
C12—C11—C10	120.8 (10)	C4—C3—C2	122.7 (9)
C16—C11—C10	120.5 (10)	C8—C3—C2	120.7 (9)
C13—C12—C11	121.0 (10)	C5—C4—C3	122.1 (9)
C13—C12—H12A	119.5	C5—C4—H4A	119.0
C11—C12—H12A	119.5	C3—C4—H4A	119.0
C12—C13—C14	121.9 (9)	C4—C5—C6	121.8 (8)
C12—C13—H13A	119.1	C4—C5—Br1	120.2 (6)
C14—C13—H13A	119.1	C6—C5—Br1	118.0 (7)
O4—C14—C13	122.7 (8)	O2—C6—C5	121.0 (8)
O4—C14—C15	120.5 (8)	O2—C6—C7	121.7 (8)
C13—C14—C15	116.8 (9)	C5—C6—C7	117.2 (8)
C16—C15—C14	122.5 (8)	C8—C7—C6	121.3 (9)
C16—C15—Br2	118.9 (7)	C8—C7—H7A	119.4
C14—C15—Br2	118.6 (7)	C6—C7—H7A	119.4
C15—C16—C11	119.2 (9)	C7—C8—C3	121.1 (9)
C15—C16—H16A	120.4	C7—C8—H8A	119.5
C11—C16—H16A	120.4	C3—C8—H8A	119.5
HWA—OW—HWB	100.5		
			
O3—C9—C10—C11	61.5 (11)	O1—C1—C2—C3	61.7 (11)
C9—C10—C11—C12	−101.5 (11)	C1—C2—C3—C4	77.6 (11)
C9—C10—C11—C16	75.1 (12)	C1—C2—C3—C8	−100.6 (11)
C16—C11—C12—C13	0.7 (14)	C8—C3—C4—C5	0.8 (14)
C10—C11—C12—C13	177.4 (9)	C2—C3—C4—C5	−177.4 (8)
C11—C12—C13—C14	0.6 (15)	C3—C4—C5—C6	0.3 (14)
C12—C13—C14—O4	178.9 (9)	C3—C4—C5—Br1	−179.2 (7)
C12—C13—C14—C15	−1.3 (13)	C4—C5—C6—O2	−178.8 (8)
O4—C14—C15—C16	−179.4 (8)	Br1—C5—C6—O2	0.7 (12)
C13—C14—C15—C16	0.8 (13)	C4—C5—C6—C7	−1.8 (13)
O4—C14—C15—Br2	0.2 (11)	Br1—C5—C6—C7	177.7 (7)
C13—C14—C15—Br2	−179.6 (6)	O2—C6—C7—C8	179.3 (8)
C14—C15—C16—C11	0.5 (14)	C5—C6—C7—C8	2.3 (14)
Br2—C15—C16—C11	−179.2 (7)	C6—C7—C8—C3	−1.2 (14)
C12—C11—C16—C15	−1.2 (14)	C4—C3—C8—C7	−0.3 (14)
C10—C11—C16—C15	−177.9 (8)	C2—C3—C8—C7	177.9 (9)

### Hydrogen-bond geometry (Å, °)

**Table:**

*D*—H···*A*	*D*—H	H···*A*	*D*···*A*	*D*—H···*A*
OW—HWA···Br1	0.89	2.86	3.537 (8)	134
OW—HWA···O2	0.89	2.00	2.820 (10)	151
O1—H1A···O4	0.82	1.84	2.633 (9)	163
OW—HWB···O1^i^	0.88	2.07	2.745 (10)	133
O2—H2A···Br1	0.85	2.56	3.026 (6)	115
O2—H2A···OW	0.85	2.18	2.820 (9)	131
O3—H3A···O2^ii^	0.82	1.80	2.592 (9)	162
O4—H4B···Br2	0.85	2.57	3.039 (7)	116
O4—H4B···OW^iii^	0.85	2.21	2.804 (10)	127

Symmetry codes: (i) *x*+1/2, *y*+1/2, *z*; (ii) *x*+1, *y*, *z*; (iii) *x*+1/2, *y*−1/2, *z*.
